# Temporal and Spatial Evolution of Vegetation Coverage in the Mianyuan River Basin Influenced by Strong Earthquake Disturbance

**DOI:** 10.1038/s41598-019-53264-5

**Published:** 2019-11-14

**Authors:** Bin-rui Gan, Xing-guo Yang, Wen Zhang, Jia-wen Zhou

**Affiliations:** 1 State Key Laboratory of Hydraulics and Mountain River Engineering, Sichuan University, Chengdu, 610065 P.R. China; 2 College of Water Resource and Hydropower, Sichuan University, Chengdu, 610065 P.R. China

**Keywords:** Evolutionary ecology, Natural hazards

## Abstract

The 2008 Wenchuan earthquake caused significant economic losses and degradation of regional ecosystems, including the terrestrial vegetation. Since the vegetation root system can enhance the soil’s anti-erosion capacity and therefore mitigate the occurrence of slope instabilities, it is beneficial to study the spatial and temporal evolution of vegetation for a long-term assessment of co-seismic secondary disasters. The Mianyuan River Basin, an uninhabited area passing through an active fault located in the earthquake-affected region, was selected as the study area. The Normal Difference Vegetation Index (NDVI) was calculated using remote sensing images from 1994 to 2017 to analyze the process of vegetation growth, loss, fluctuation and recovery. Statistical results suggest that the area in the middle and lower reaches, near the river network, and with a slope of 30 to 40 degrees were variable regions, showing more significant vegetation destruction during the earthquake and faster repair after the seismic event. Besides, vegetation near the fault was damaged more severely after the earthquake, but the active fault did not play an essential role in the vegetation recovery period. In the Mianyuan River Basin, vegetation experienced a volatility period (5 plus or minus one year) before entering the recovery period. In 8 to 9 years after the earthquake, the surficial vegetation could recover to the state before the earthquake.

## Introduction

**Figure 1:**
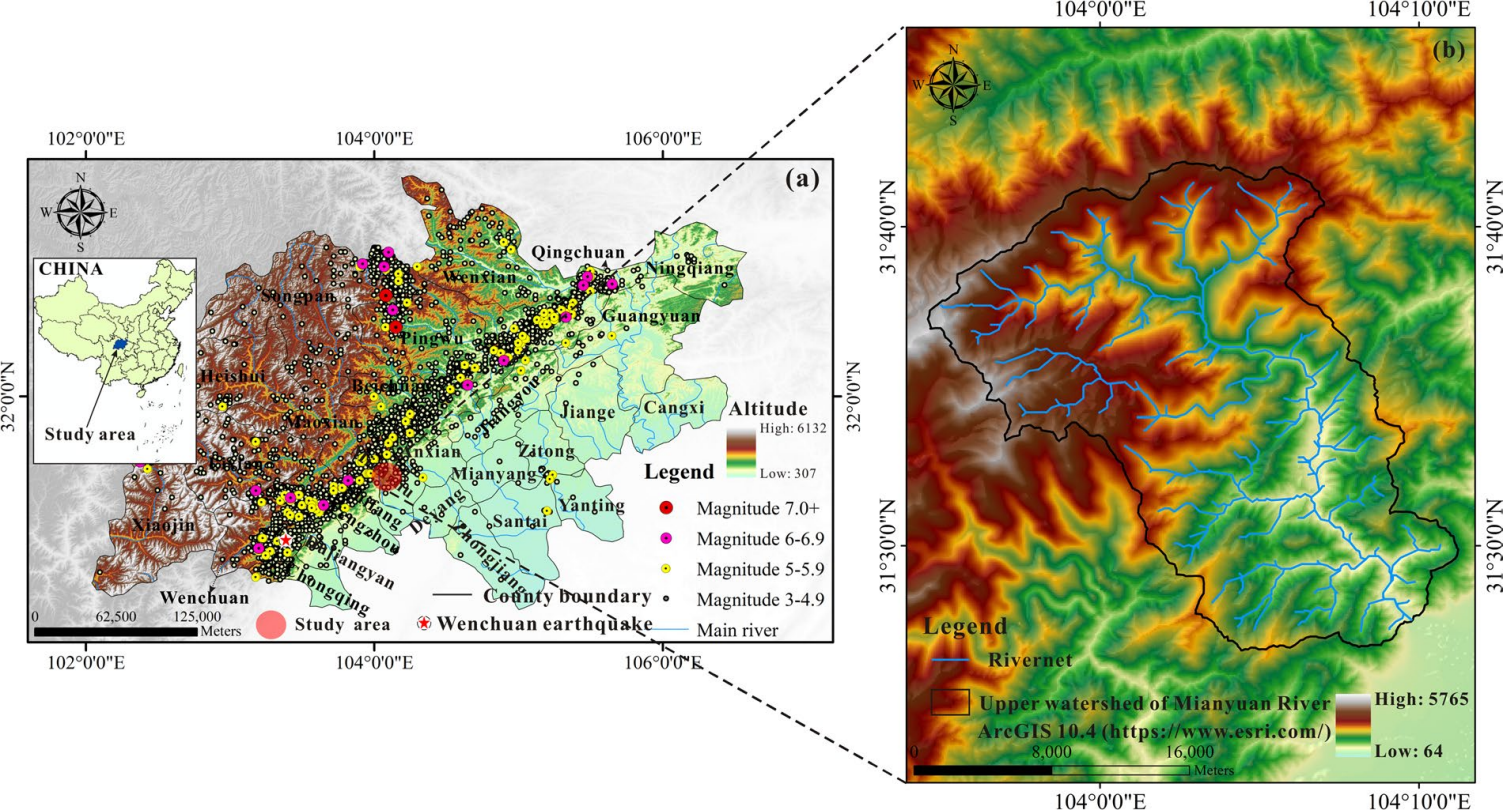
Seismic statistics near the Longmen Shan Fault (FLZ) Zone (**a**) and the overall of the study area (**b**).

The strong tectonic movement caused the Qinghai-Tibet Plateau uplift and formed the Longmenshan Fault^1–3^. The notorious fault has nurtured some of the most catastrophic earthquakes in human history, including the Wenchuan earthquake on May 12, 2008 (Fig. 1a). On that day, the tectonic stress accumulated over long periods of geological time released abruptly in the area between Beichuan and Yingxiu (epicenter at a north latitude of 31.01° and east longitude of 103.42°), triggering great abundant of vegetation loss together with co-seismic landslides^4–8^. Since the vegetation and its root systems can prevent soil erosion and reinforce the slopes, the earthquake-affected areas became more prone to secondary disasters such as mountain floods, debris flow and landslides, which threatening the property and safety of local residents^9–11^. Therefore, whether the vegetation will recover spontaneously or even deteriorate is worth studying. The Chinese government attaches great importance to the post-earthquake hazards and has invested a large amount of funds for disaster prevention and mitigation. Understanding the vegetation evolution after the strong earthquake is therefore essential, and could provide the government with key time points for vegetation recovery, shortening the duration of disaster prevention^9^.

With the rapid development of space platforms and digital imagery, rational interpretation of remote sensing data can thus facilitate efficient natural-hazard investigation and management^12^. In previous studies, the effects of altitude, fault zone, seismic intensity, soil texture, vegetation type, distance from river network, and slopes on vegetation recovery had been preliminarily and separately studied. For instance, Jiang *et al*. (2015) studied the variation of vegetation cover in the Longmenshan area and concluded that vegetation recovery was faster at higher altitudes^13^. Lu *et al*. (2010) presented the relationship between several spatial characteristics and vegetation loss^9^. Liu *et al*. (2017) interpreted the remote sensing images of the entire Wenchuan earthquake-affected area to get vegetation recovery rate in 2016 and estimated the time for complete vegetation recovery^14^. It is over ten years after the catastrophic Wenchuan earthquake as of today. Examination of the vegetation recovery in a specific time period can be insufficient to understand the recovery of the ecosystem. Therefore, the spatial and temporal evolution of vegetation in the earthquake-stricken areas is scrutinized in this study.

Vegetation loss and the frequency of secondary disasters have an interactive relationship. It is possible that the vegetation loss after the earthquake aggravated the frequency of secondary disasters, resulting in continuous damage to the vegetation and then forming a vicious circle. It is also possible that vegetation may also recover relatively quickly and the frequency of secondary disasters reduced gradually, and thus forming a virtuous cycle of ecological restoration in earthquake-affected areas. To better understand the dynamic process of post-earthquake vegetation evolution, areas with less human activity rather than the whole earthquake-affected areas should be used as the study area.

In this study, the Normal Different Vegetation Index (NDVI) was used to illustrate the surficial vegetation coverage condition in an uninhabited area affected by the 2008 Wenchuan earthquake. Remote sensing imageries and Geographic Information System (GIS) were used to calculate the NDVI before and after the Wenchuan earthquake. After that, we demonstrated a relatively balance in the fluctuation of vegetation before the earthquake and vegetation loss and recovery after the earthquake in detail. Meanwhile, the effects of topographic characteristics and distance from the river network on vegetation were also studied. Besides, the temporal and spatial evolution of vegetation loss and natural recovery in the earthquake-affected area were discussed, and the key time points of volatility and recovery periods were indicated.

## Background

**Figure 2:**
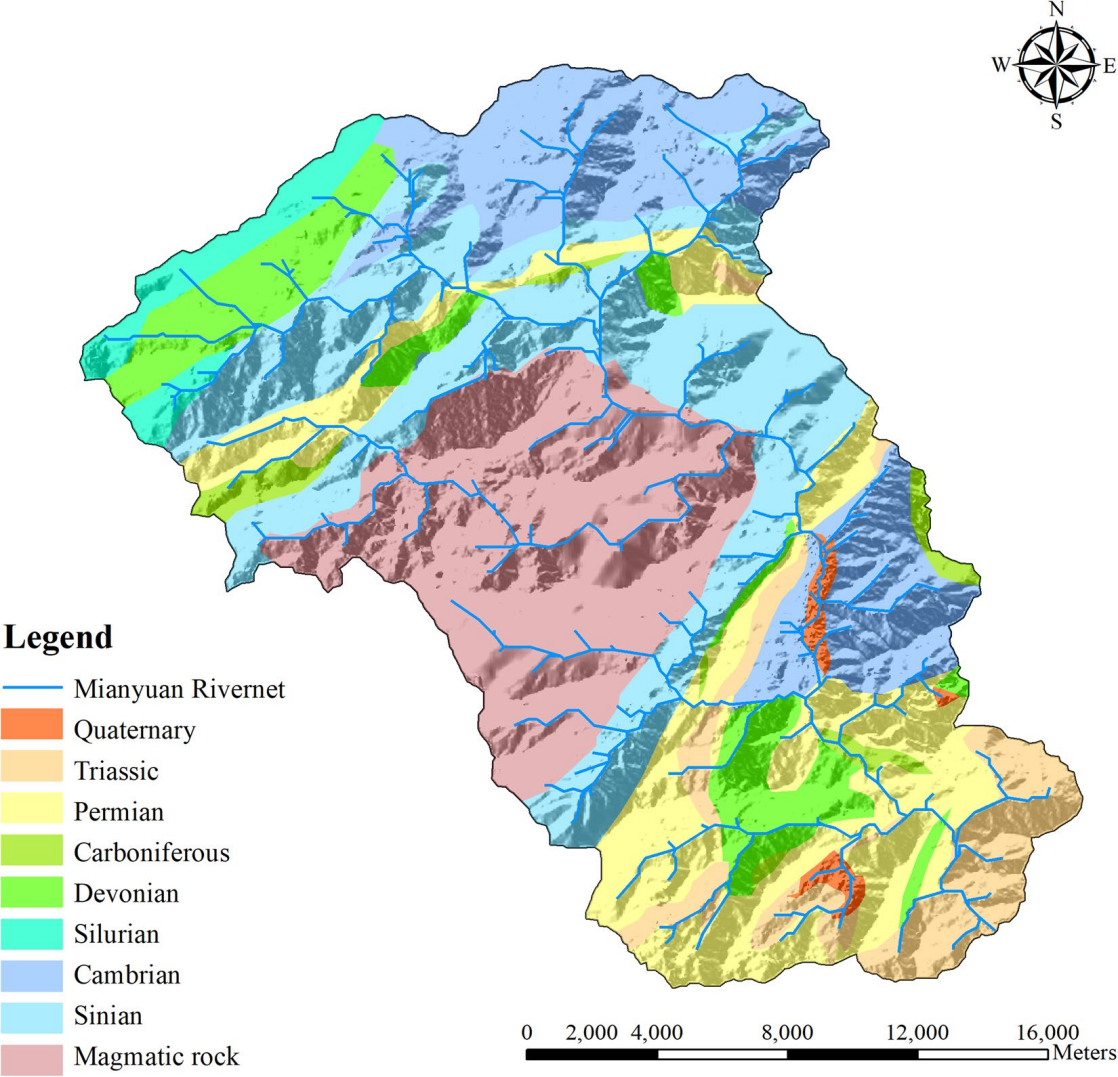
Geological setting of the Mianyuan River Basin.

**Figure 3:**
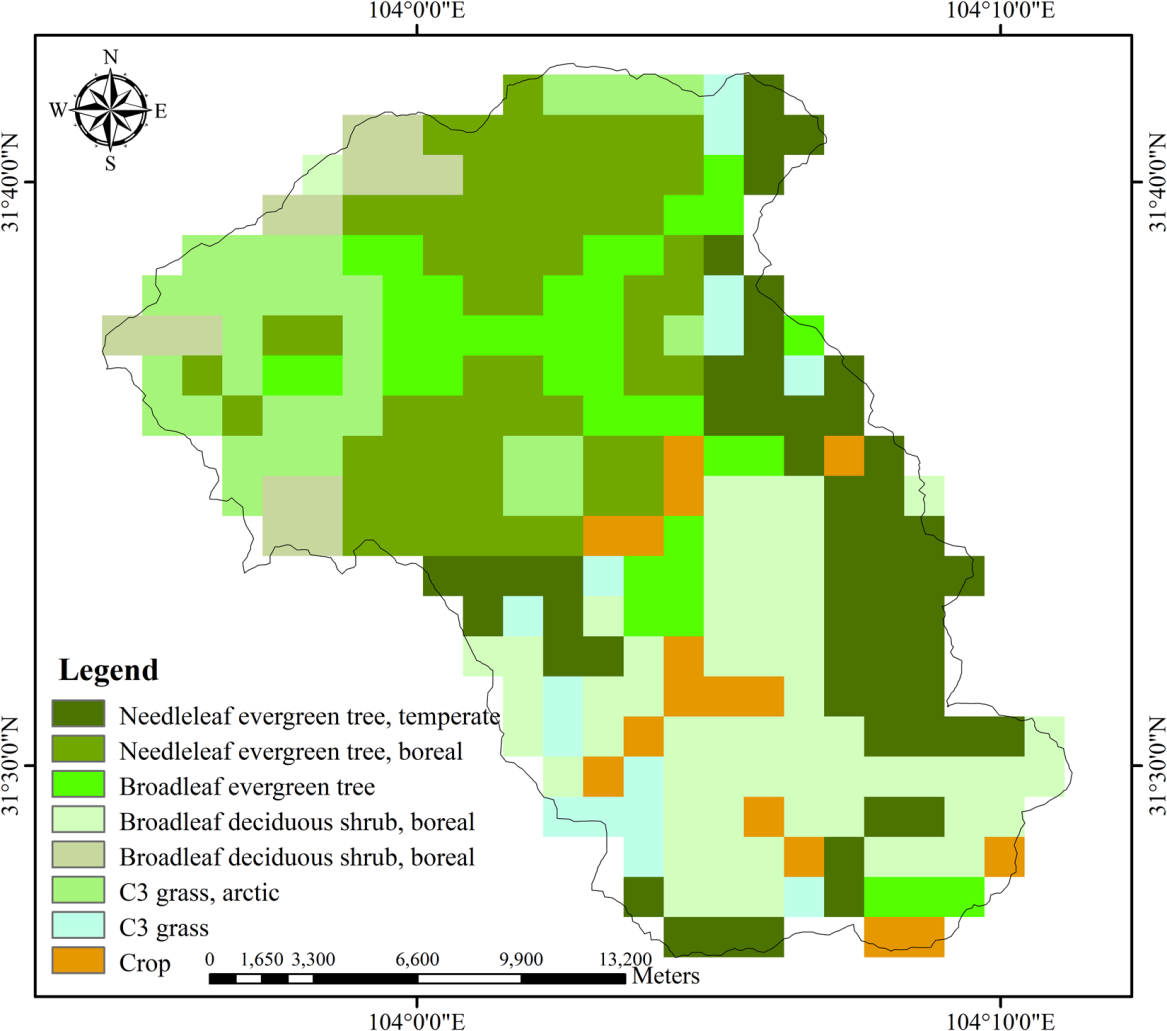
Overview of Vegetation Types in the Mianyuan River Basin.

The Mianyuan River basin (Fig. 1b) is an uninhabited area located in Mianzhu County, southwest China, and the Mianyuan River origins from the north of the Jiuding Shan Mountains. The basin is about 80 km northeast of the Wenchuan earthquake epicenter, and a substantial amount of co-seismic disasters (landslide dams, debris flows) were induced by the earthquake, leading to significant ecological degradation^15^. Human factors such as construction and reclamation have negligible effects on the natural ecological environment in the basin, and it is thus selected as the study area^16,17^. Figure 2 shows the geological setting of this basin^16^. The outcrops within the basin show the Sinian, Cambrian, Silurian, Devonian, Carboniferous, Permian and Triassic rocks, which are covered by loose Quaternary materials. The area of the Mianyuan River Basin is about 401 km^2^ with the highest elevation about 4,417 m and the lowest about 697 m^18^. The Yingxiu-Beichuan fracture (mid-fracture) of the Wenchuan earthquake goes through the middle part of the Mianyuan River Basin. The southwest mountainous area has a subtropical monsoon climate, with hot and rainy summers and less moderate precipitation in winter. The annual rainfall is between 1,500 to 1,700 mm and mainly concentrated in July-September^18^. Figure 3 shows the vegetation types in the Mianyuan River Basin (This data set is provided by Cold and Arid Regions Science Data Center at Lanzhou (http://westdc.westgis.ac.cn)^19^. As shown in Fig. 3, the vegetation types in the Mianyuan River Basin are mainly Needleleaf evergreen tree, Broadleaf evergreen tree, Broadleaf deciduous tree and grass, most of which are evergreen.

## Method

**Figure 4:**
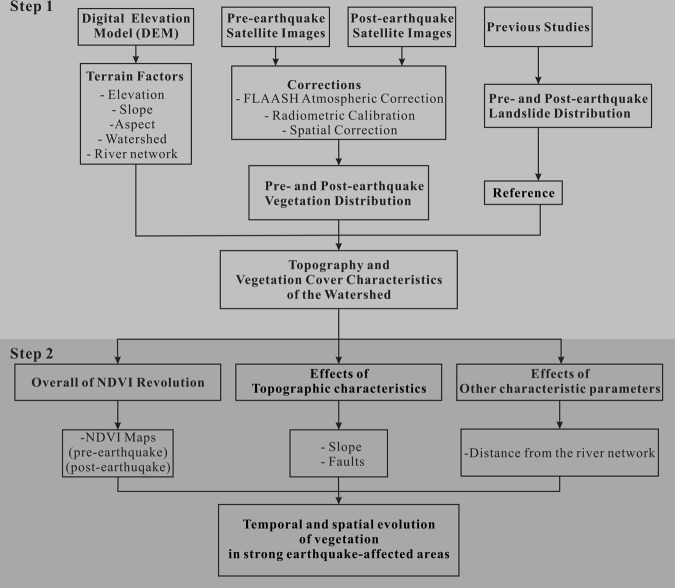
Flowchart of research tasks implemented in this paper.

**Figure 5:**
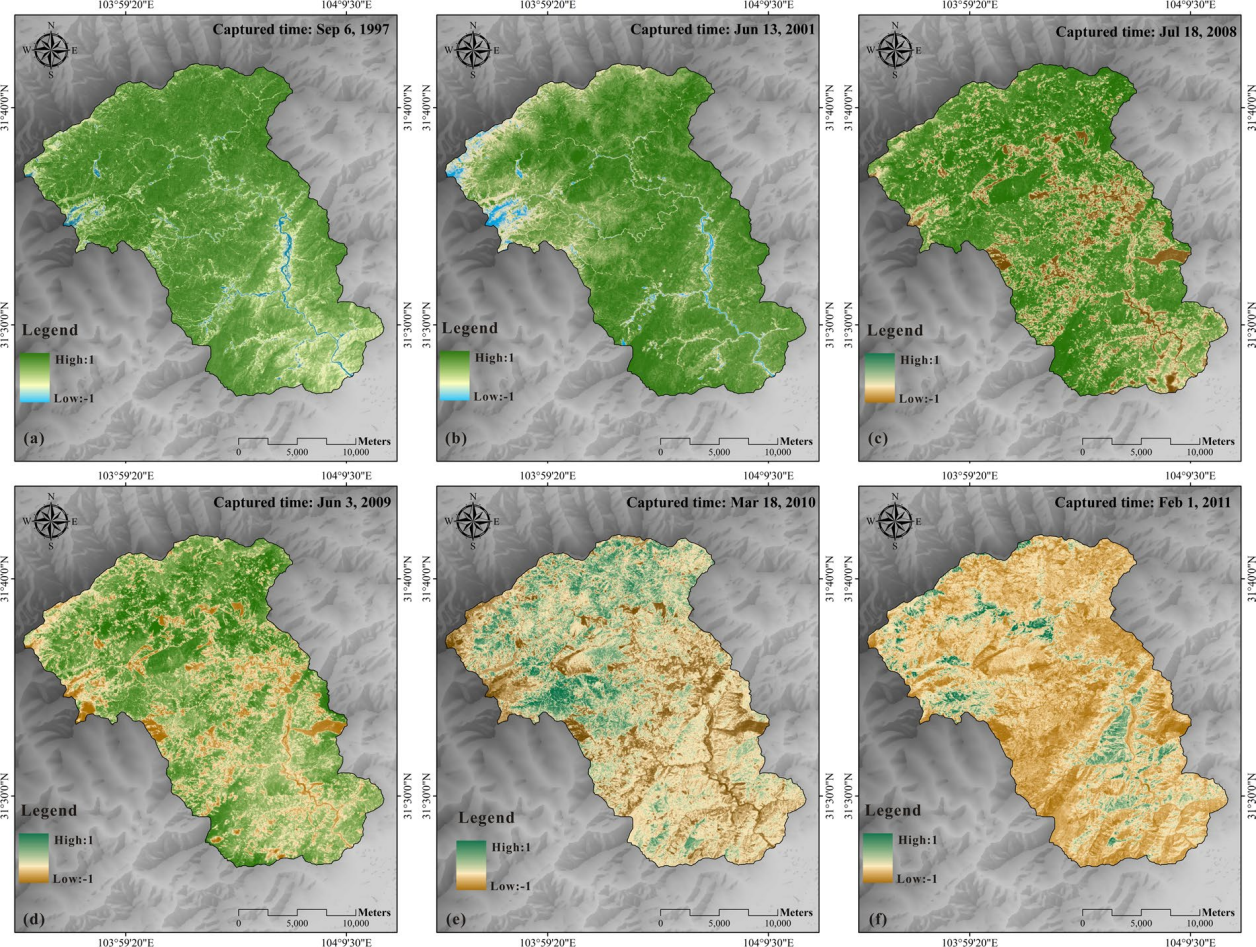
Normal Difference Vegetation Index (NDVI) maps of the Mianyuan River Basin calculated by Landsat 5: (**a**) NDVI map in 1997, (**b**) NDVI map in 2001, (**c**) NDVI map in 2008, (**d**) NDVI map in 2009, (**e**) NDVI map in 2010 and (**f**) NDVI map in 2011.

In this study, three different datasets were used: (1) Pre-earthquake and post-earthquake satellite image data (Table 1). These images were mainly captured at every summer to avoid snow cover. (2) Several Digital Elevation Models (DEM) of the earthquake-affected area downloaded from Geospatial Data Cloud (http://www.gscloud.cn/). The elevation value of DEM was from 307 to 6132 meters. (3) Scientific reports, papers and maps related to landslides, vegetation and fault distribution in the Wenchuan earthquake-affected areas. In addition, the method used in this research, as shown in Fig. 4, comprised two main steps. The Original data for the Figs 1, 2, 5, 7, 9, 10 and 11 were obtained from Geospatial Data Cloud (http://www.gscloud.cn/, Free Access for use) and ArcGIS 10.4 (https://www.esri.com/) was used to create the images.

**Table 1:** Satellite images data index table.

Id	Date	Sensor	Resolution (m)	Source	Type of data	Production ID
**Pre-earthquake**						
1	06/26/1994	TM	30	Landsat 5	L45TM	LT51300381994177BKT00
2	08/25/1995	TM	30	Landsat 5	L45TM	LT51290381995237CLT00
3	07/10/1996	TM	30	Landsat 5	L45TM	LT51290381996192CLT00
4	07/17/1996	TM	30	Landsat 5	L45TM	LT51300381996199BKT00
5	09/06/1997	TM	30	Landsat 5	L45TM	LT51300381997249BKT00
6	06/13/2001	TM	30	Landsat 5	L45TM	LT51300382001164BJC00
7	10/28/2001	TM	30	Landsat 5	L45TM	LT51290382001301BJC00
8	12/22/2001	TM	30	Landsat 5	L45TM	LT51300382001356BJC00
9	01/07/2002	TM	30	Landsat 5	L45TM	LT51300382002007BJC00
10	04/06/2002	TM	30	Landsat 5	L45TM	LT51290382002096BJC00
11	07/11/2002	TM	30	Landsat 5	L45TM	LT51290382002192BJC00
12	05/21/2004	ETM+	30	Landsat7	L7slc-off	L71129038_03820040521
13	09/09/2004	TM	30	Landsat 5	L45TM	LT51300382004253BJC00
14	03/05/2005	ETM+	30	Landsat7	L7slc-off	L71129038_03820050305
15	05/19/2006	ETM+	30	Landsat7	L7slc-off	L5129038_03820060519
16	01/06/2007	ETM+	30	Landsat7	L7slc-off	L71129038_03820070106
17	04/19/2007	ETM+	30	Landsat7	L7slc-off	L71130038_03820070419
18	09/19/2007	ETM+	30	Landsat7	L7slc-off	L71129038_03820070919
19	04/30/2008	ETM+	30	Landsat7	L7slc-off	L71129038_03820080430
**Post-earthquake**						
1	07/18/2008	TM	30	Landsat 5	L45TM	LT51300382008200BKT00
2	10/23/2008	ETM+	30	Landsat7	L7slc-off	LE71290382008297EDC00
3	12/01/2008	ETM+	30	Landsat7	L7slc-off	L71130038_03820081201
4	03/15/2009	TM	30	Landsat 5	L45TM	LT51300382009074BKT00
5	03/16/2009	ETM+	30	Landsat7	L7slc-off	L71129038_03820090316
6	03/24/2009	TM	30	Landsat 5	L45TM	LT51290382009083BJC00
7	05/03/2009	ETM+	30	Landsat7	L7slc-off	L71129038_03820090503
8	06/03/2009	TM	30	Landsat 5	L45TM	LT51300382009154BJC00
9	06/12/2009	TM	30	Landsat 5	L45TM	LT51290382009163BJC00
10	03/10/2010	ETM+	30	Landsat7	L7slc-off	L71130038_03820100310
11	03/18/2010	TM	30	Landsat 5	L45TM	L5130038_03820100318
12	02/01/2011	TM	30	Landsat 5	L45TM	LT51300382011032BKT00
13	08/05/2011	TM	30	Landsat 5	L45TM	L5129038_03820110805
14	03/27/2013	ETM+	30	Landsat7	L7slc-off	LE71290382013086EDC00
15	08/18/2013	ETM+	30	Landsat7	L7slc-off	LE71290382013230PFS00
16	02/01/2014	ETM+	30	Landsat7	L7slc-off	LE71300382014032EDC00
17	04/02/2015	ETM+	30	Landsat7	L7slc-off	LE71290382015092EDC01
18	02/07/2016	ETM+	30	Landsat7	L7slc-off	LE71300382016038EDC00
19	05/06/2016	ETM+	30	Landsat7	L7slc-off	LE71290382016127NPA00
20	06/07/2016	ETM+	30	Landsat7	L7slc-off	LE71290382016159EDC00
21	09/11/2016	ETM+	30	Landsat7	L7slc-off	LE71290382016255EDC00
22	04/14/2017	ETM+	30	Landsat7	L7slc-off	LE07_L1TP_130038_20170414_20170510_01_T1

In the first step, various types of watershed information including river network in the Mianyuan River Basin were calculated using the collected DEM data. Then, the pre- and post-earthquake satellite images were used to calculate the NDVI before and after the earthquake to illustrate the surficial vegetation coverage^20,21^. The NDVI was expressed as follows:

1 $${\rm{NDVI}}=\frac{{\rm{NIR}}-{\rm{R}}}{{\rm{NIR}}+{\rm{R}}}$$

where NIR is the reflectance value at the near infrared band, and R is the reflectance value at the red band. If the NDVI is less than 0, the ground cover is a high reflection of visible light such as clouds, water, and snow. If NDVI is equal to 0, it means that there are rocks or bare soil. If NDVI is greater than zero, it shows vegetation coverage and the value increases with coverage. All satellite images were processed with atmospheric correction, radiation correction and spatial correction before NDVI calculations.

In the second step, NDVI maps at different times were created to illustrate the details of vegetation distribution before and after the earthquake. In addition, differences between the NDVI data of two consecutive years (Landsat 5-TM) were subtracted to get the vegetation changes in the earthquake-affected area. Meanwhile, effects of topographic characteristics on vegetation loss and recovery were discussed. Other characteristic parameters (such as the distance from the river) which may directly or indirectly affect the revolution of vegetation were discussed as well. After that, temporal and spatial evolution of vegetation under the influence of external factors in the earthquake-affected area and the key time points of volatility and recovery periods were demonstrated.

## Results

### Vegetation distribution maps

**Figure 6:**
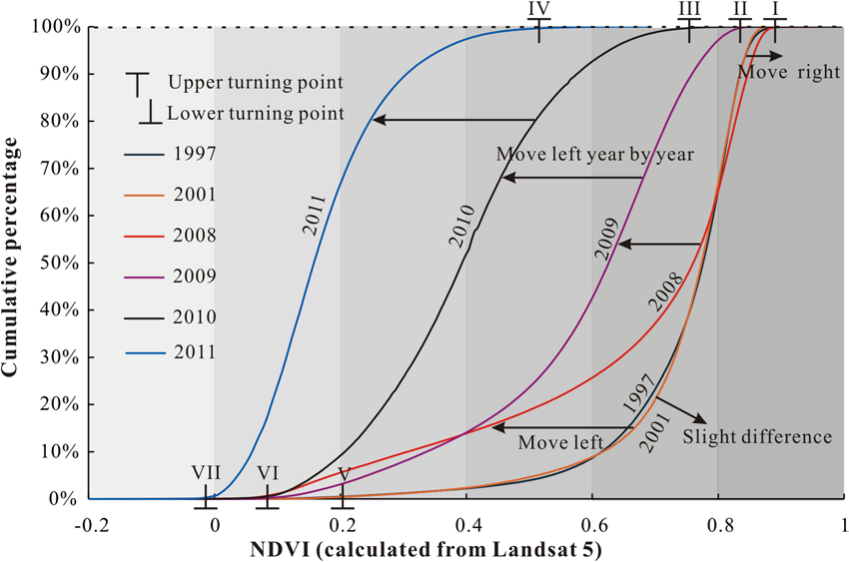
NDVI cumulative curve calculated using Landsat 5.

The combination of the Wenchuan earthquake in 2008 and other exogenic factors (e.g., snowmelt, heavy rainfall) made the studied mountainous area highly vulnerable to landslide hazards, disturbing vast area of vegetation^1,22–24^. Thus, the vegetation coverage in the earthquake-affected area is subject to dynamic changes. Figure 5 illustrates the evolution of the NDVI distribution in the Mianyuan River Basin calculated from Landsat 5-TM data. Among them, the NDVI distribution maps of several years were abnormal because of cloud occlusion. Some areas were covered with snow all year round because of the high altitudes. As shown in Fig. 5, the surficial vegetation coverage before the earthquake exhibited relative balance with fluctuations (Fig. 5a and Fig. 5b) whereas it was seriously damaged after the Wenchuan earthquake (Fig. 5c–f). Figure 6 presents the cumulative percentage of NDVI. These curves are S-shaped curves with upper and lower turning points. The lower turning point refers to the point when the cumulative percentage of NDVI changes from 0% to a value. The upper turning point refers to the point when the cumulative percentage of NDVI rises to a certain stage and becomes stable. The larger the NDVI value at the upper inflection point, the higher the vegetation coverage rate, and the better the surficial vegetation coverage. The greater the value of NDVI at the lower turning point, the lower the vegetation coverage rate and the better the surficial vegetation coverage. As shown in Fig. 6, the upper and lower turning points of the cumulative curve of NDVI remained unchanged from 1997 to 2001 except for slight differences in curvature. After seven years, the Wenchuan earthquake occurred and the upper tuning point of the NDVI curve moved right slightly while the lower turning point moved left. Since 2008, the NDVI curve has gradually shifted to the left year after year with decreases in upper and lower turning points.

**Figure 7:**
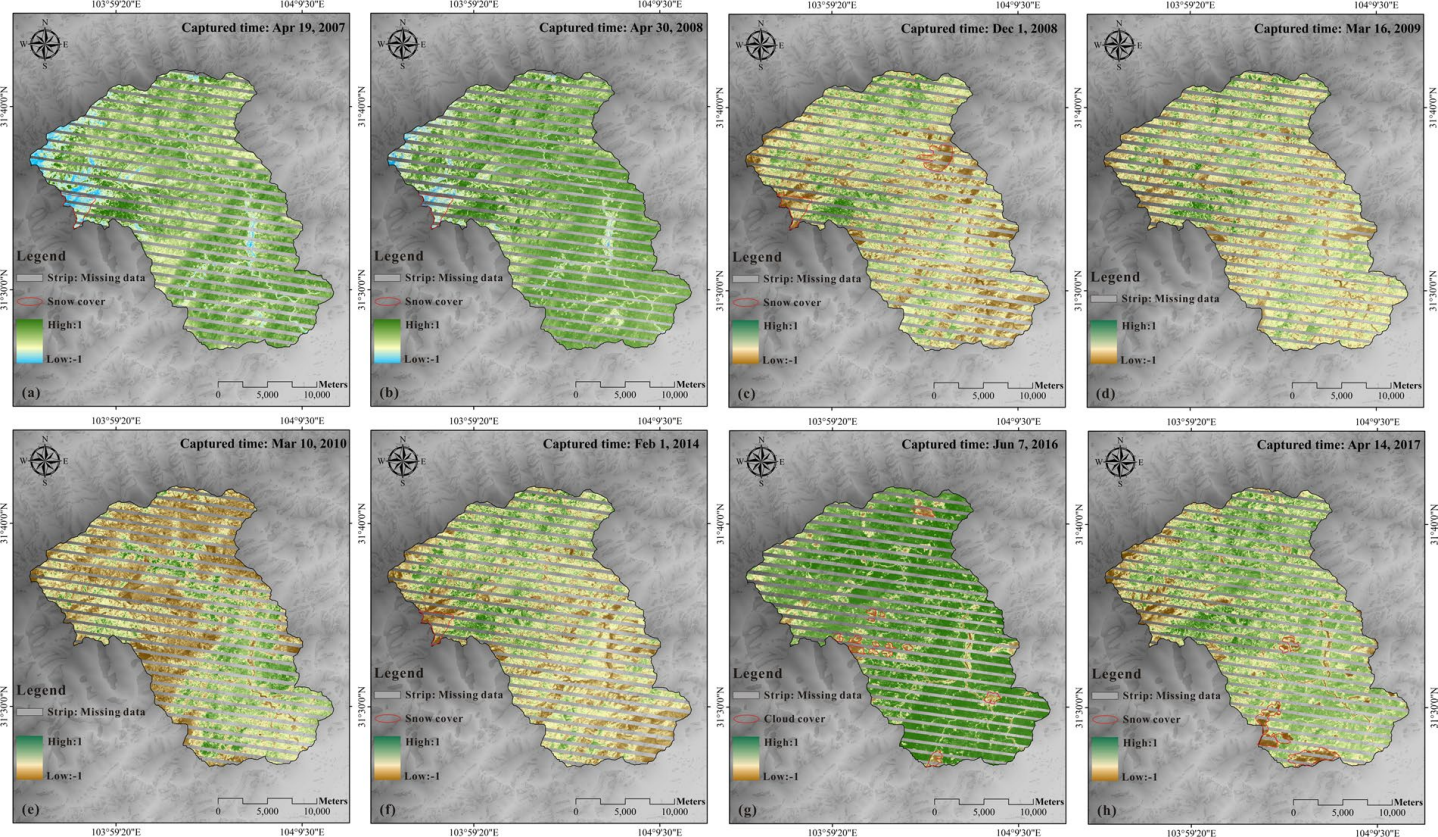
Normal Difference Vegetation Index (NDVI) maps of the Mianyuan River Basin calculated by Landsat 7: (**a**) NDVI map in 2007, (**b**) NDVI map in Apr 30, 2008, (**c**) NDVI map in Dec 1, 2008, (**d**) NDVI map in 2009, (**e**) NDVI map in 2010, (**f**) NDVI map in 2014, (**g**) NDVI map in 2016 and (**h**) NDVI map in 2017.

**Figure 8:**
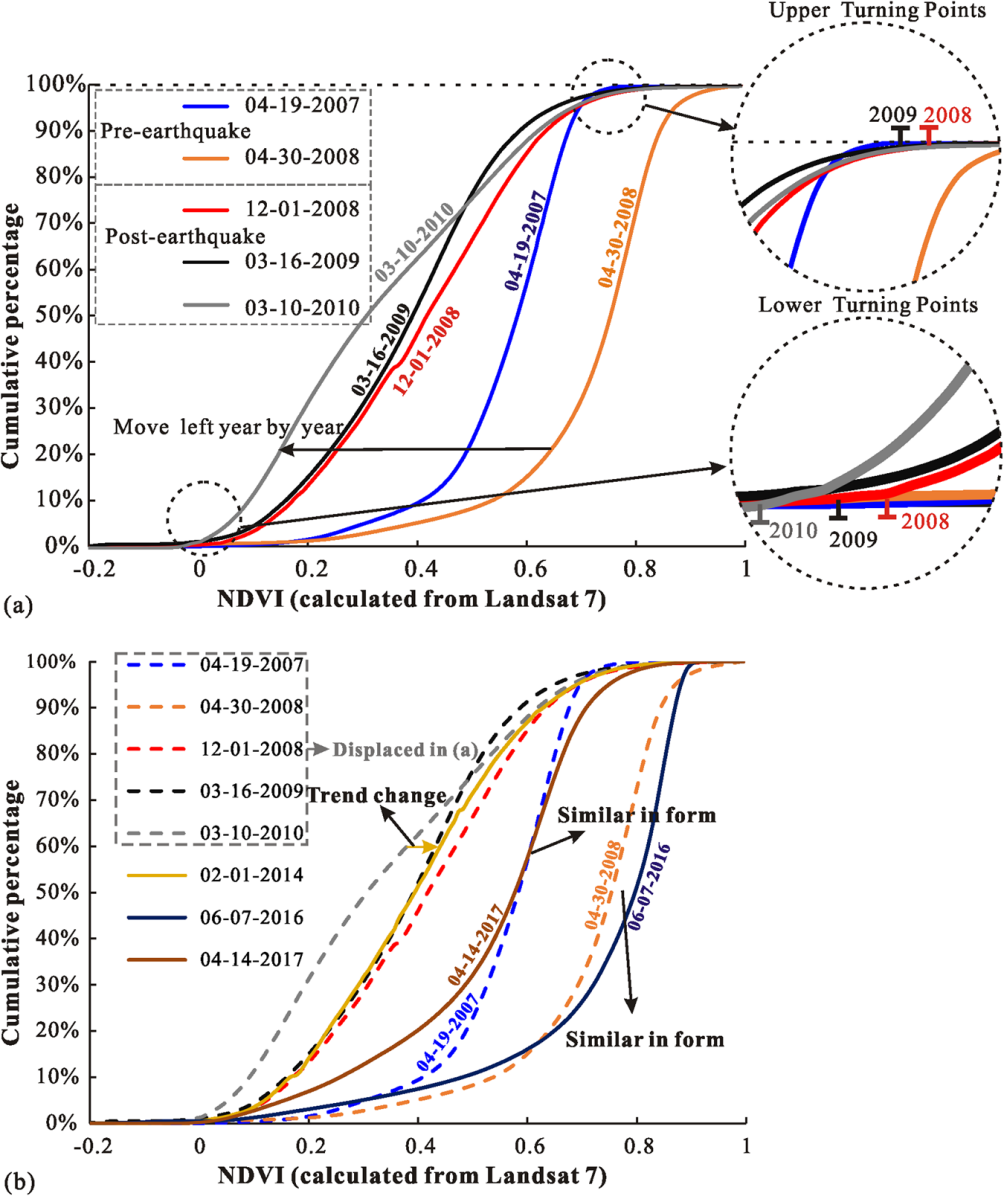
NDVI cumulative curve calculated using Landsat 7.

Vegetation in the earthquake-affected areas was also subject to slow natural recovery after the Wenchuan earthquake. The NDVI distribution maps before and after the shock in the Mianyuan River Basin were calculated by the Landsat 7-ETM+satellite images. The NDVI values calculated by Landsat 5 and Landsat 7 were not analogous because of the use of different sensors. As shown in Fig. 7, owing to the failure of the Landsat 7-ETM+ onboard scan line corrector, the collected image had data strips losses. Therefore, only part of the data was processed and compared. Despite this, remote sensing data from nearly the same month was used to eliminate the difference between the bands. From the NDVI distribution maps, it appears that the vegetation loss due to the disturbances from 2008 Wenchuan earthquake gradually recovered to the pre-earthquake status approximately in 2016. Figure 8 shows the statistics of NDVI changes. It indicates that the NDVI curve shifted to the right before the earthquake, representing a gradual increase in vegetation. The NDVI curve after the quake showed a robust shift to the left. The upper and lower turning points of the NDVI curve in 2009 were on the left side of 2008, which indicated that the area of high surficial vegetation coverage in 2009 decreased compared with that in 2008 whereas the low vegetation area increased. Not only that, the area of low surficial vegetation coverage in 2010 was significantly higher than that of 2009 (the turning point of the NDVI curve in 2010 shifted significantly to the left relative to that of 2009). In addition, the curve of 2014 began to move to the right compared to 2010, indicating that the vegetation gradually became dense after the earthquake (Fig. 8b). The NDVI curve in 2016 was similar to the pre-earthquake curve (April 30, 2008), and the 2017 NDVI curve was identical to the curve on April 19, 2007.

Figure 8a presents the slight right shift of NDVI curves before the earthquake and the left shift of NDVI curves after the earthquake, which shows the same phenomenon as NDVI calculated from Landsat 5. Furthermore, the curve in 2014 began to shift to the right compared to that in 2010, indicating the change in the trend of NDVI after the earthquake (Fig. 8b). After 2010, the NDVI curve shifted to right gradually. Moreover, the 2016 NDVI curve was similar to the curve before the earthquake (Apr. 30, 2008) and the 2017 NDVI curve was similar to the curve in Apr. 19, 2007.

### NDVI difference

**Figure 9:**
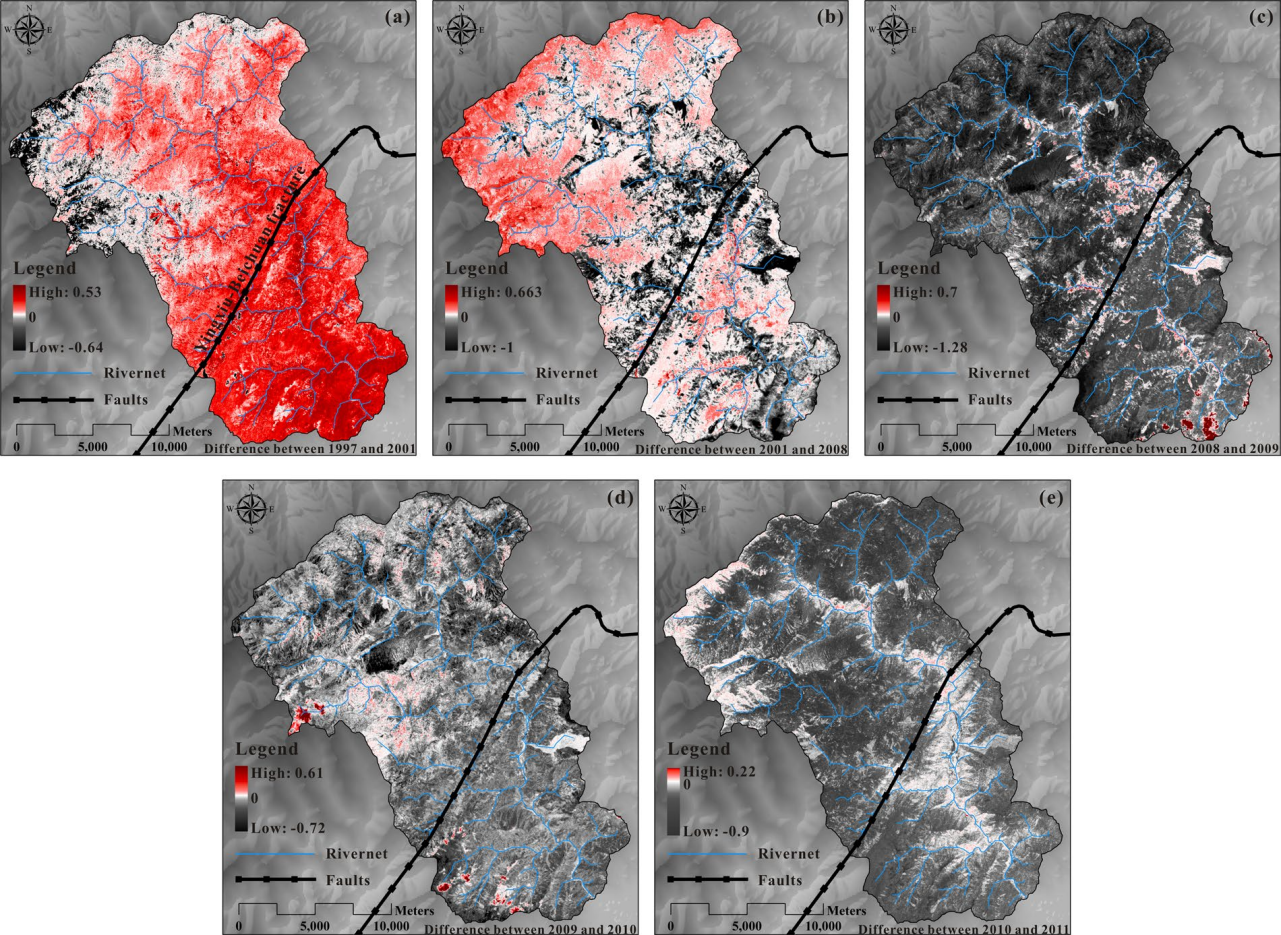
NDVI difference distribution map: (**a**) NDVI changes from 1997 to 2001, (**b**) NDVI changes from 2001 to 2008, (**c**) NDVI changes from 2008 to 2009, (**d**) NDVI changes from 2009 to 2010 and (**e**) NDVI changes from 2010 to 2011.

The surficial vegetation coverage in earthquake-affected areas has been described above. From Figs. 5 and 7, there are also differences in the conditions affected by earthquakes in various areas of the basin. The NDVI values in two consecutive years were subtracted to get a distribution map of vegetation differences (Fig. 9). As shown in Fig. 9a, due to winter snow cover, the NDVI in the plateau region was reduced. Furthermore, comparison analysis the surficial vegetation coverage between 2001 and 1997 indicated that, the surficial vegetation coverage in the middle and lower reaches of the basin increased more significantly. Several years after the catastrophic earthquake, surficial vegetation coverage in the Mianyuan River Basin was greatly disturbed (Fig. 9b). In particular, the surficial vegetation coverage in the middle and lower reaches of the basin and the near-river area was substantially reduced. However, surficial vegetation coverage in high altitude areas shows an increasing trend. Since then, as shown in Fig. 9c, the surficial vegetation coverage in 2009 showed a weakening trend compared to the previous year, but signs of slow repair began to appear in the near-river area. But one year later (in 2010, as shown in Fig. 9d), the surficial vegetation coverage of the entire basin became weakened overall and this trend continued until 2011 (Fig. 9e).

The main factors affecting vegetation recovery include three aspects: environmental factors (air, humidity and others), soil nutrient factors (soil nitrogen, TVDI, organic carbon and others) and topographical factors (slope, aspect and others). The Mianyuan River Basin is an uninhabited area, and thus the environmental factors and soil nutrient factors cannot be comprehensively measured. Therefore, this study mainly focuses on the impact of topographic factors on vegetation changes. After the earthquake, the loss of vegetation near the fault was more serious (Fig. 9b). The larger distance away from the fault, the smaller the disturbance of the vegetation. As the lower reaches of the Mianyuan River Basin were close to the Dujiangyan-Hanwang fault, the vegetation damage was also serious. Moreover, as shown in Fig. 9c–e, faults did not have much impact on vegetation recovery.

### Effects of topographic characteristics

**Figure 10:**
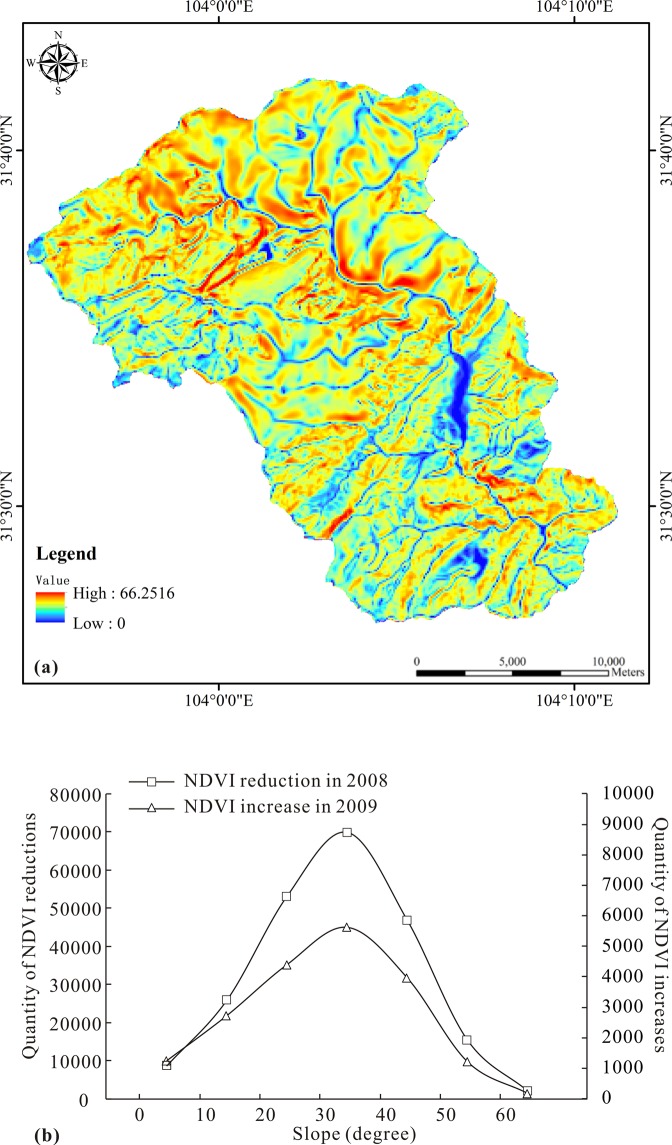
The relationship between slope and vegetation loss and recovery: (**a**) slope distribution map of the Mianyuan River Basin and (**b**) relationship between slope and vegetation loss and recovery.

Slope gradient is a key topographic factor that affects vegetation changes. Figure 10a shows the slope distribution of the Mianyuan River Basin calculated from the digital elevation model. The slope distribution map is an overlap with the pre-earthquake growth and post-earthquake loss of vegetation, and the relationship between the vegetation changes and slopes is obtained with and without earthquake disturbance. As shown in Fig. 10b, earthquakes have different effects on vegetation for different slopes. Specifically, when the slope is less than 30 degrees, the vegetation coverage reduces when the slope increases. When the slope exceeds 40 degrees, the disturbance of vegetation coverage is gradually reduced. After the strong influence of earthquakes, the surficial vegetation coverage of different slopes also showed different evolution trends. As shown in Fig. 9b, although the earthquake caused a lot of vegetation losses and could not recover to the pre-earthquake situation in a short term, the vegetation still recovered in a few areas of the Mianyuan River Basin. In particular, vegetation recovered more easily for slopes between 30 degrees to 40 degrees. Overall, the relationship between the vegetation changes and slopes is consistent, and the vegetation on slopes at 30–40 degrees is more susceptible to disturbances.

**Figure 11:**
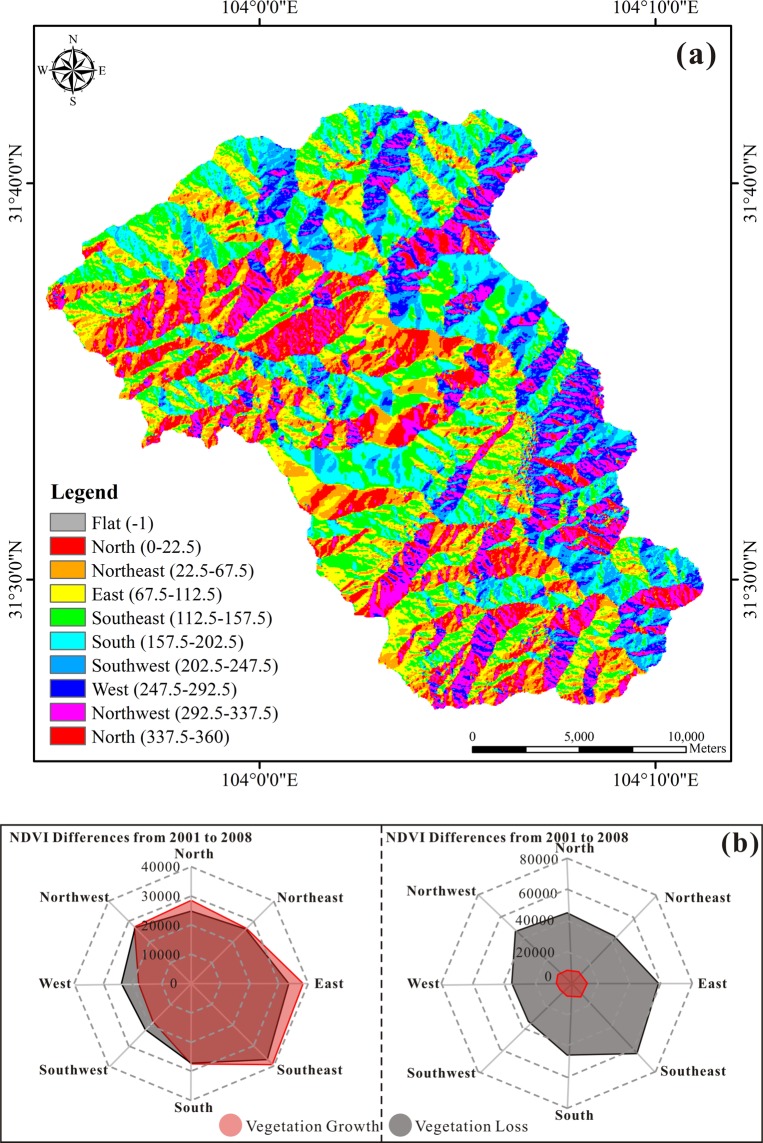
The relationship between aspect and vegetation loss and recovery: (**a**) aspect distribution map of the Mianyuan River Basin and (**b**) relationship between aspect and vegetation loss and recovery.

Aspect is another topographical feature that affects vegetation growth. Differences in conditions such as lighting lead to differences in vegetation growth rates. Figure 11a shows the aspect distribution of the Mianyuan River Basin calculated by the digital elevation model. The aspect distribution map is a spatial joint with the pre-earthquake growth and post-earthquake loss of vegetation, and the relationship between vegetation change and aspect is thus obtained as shown in Fig. 11b. From 2001 (before the earthquake) to 2008 (after the earthquake), the vegetation loss in the Mianyuan River Basin was very obvious. In particular, the vegetation in the southeast decreased the most, and those in the west and southwest decreased the least. During this time, the growth of vegetation continued, and the trend of vegetation growth was almost consistent with the trend of vegetation loss. One year after the earthquake, vegetation loss was more serious while vegetation recovery was less evident. The vegetation in the southeast and east reduced most. The recovery of vegetation is similar in all directions.

### Effects of distance from river network

**Figure 12:**
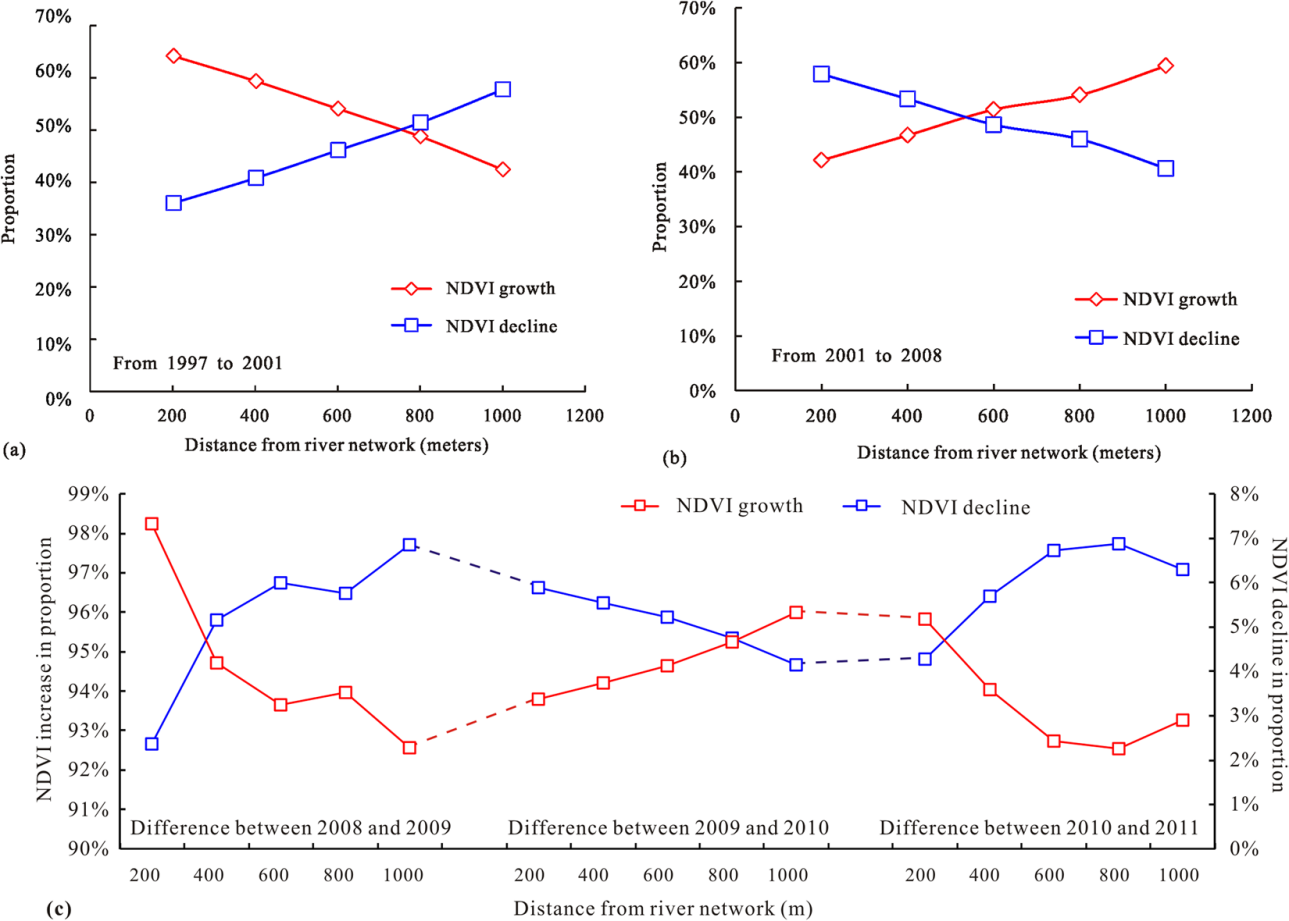
Relationship between NDVI variation and the distance from river network: (**a**) NDVI variation from 1997 to 2001, (**b**) NDVI variation from 2001 to 2008, (**c**) NDVI variation from 2008 to 2009, from 2009 to 2010 and from 2010 to 2011.

The details of the NDVI change were obtained by subtracting the NDVI distribution data for several years (as shown in Fig. 9). To observe the growth and loss of vegetation coverage in different regions more intuitively, a multi-buffer distance of 200 meters, 400 meters, 600 meters, 800 meters and 1000 meters from the river network was established, and the proportions of NDVI greater than zero (NDVI growth) and less than zero (NDVI decline) in each buffer were counted. As shown in Fig. 12a, from 1997 to 2001, the increasing ratio of NDVI decreased with the increase of the distance from the river network, while the decreasing ratio of NDVI showed the opposite trend. After the Wenchuan earthquake, from 2001 to 2008 (Fig. 12b), the trend of NDVI changes was opposite to that from 1997 to 2001. It means that the closer to the river, the greater the disturbance of surficial vegetation coverage under the disturbance of the earthquake. Within a few years after the earthquake, the vegetation in the earthquake-affected area experienced a period of volatility. During this period, the growth and loss of vegetation showed irregularity (Fig. 12c).

By establishing multi-buffer zones to analyze the increase and decrease of NDVI, the evolution trend of vegetation coverage in earthquake-affected areas is obtained. Without the seismic disturbances, the farther away the river network is, the faster the vegetation grows. When the earthquake occurs, the vegetation near the river will be more disturbed. In the years after the earthquake, the ecological environment of the earthquake-affected zone will undergo a volatile period.

## Discussions

### Impact of the earthquake on vegetation

Strong earthquakes have a complex and long-term impact on the ecosystem by changing the original landscape and destroying terrestrial vegetation, which in turn increase the possibility of secondary disasters (debris flows, dammed lakes)^25,26^. It also increases the complexity of vegetation recovery and the vulnerability of the ecological environment in the affected area. Factors such as human activities and heavy rainfall can also disturb the ecology in the Mianyuan River Basin continually. Before the earthquake, the ecological environment of the Mianyuan River Basin maintained a relatively balanced state despite the external disturbances. The fact that the vegetation did not decrease before the earthquake confirmed this. After the earthquake, due to the fragility and sensitivity of the ecological environment, the external disturbances can have a greater impact on the basin, and the surficial vegetation coverage of the Mianyuan River Basin thus reduced significantly. As shown in Fig. 9b–e, the surficial vegetation coverage decreased on a large scale for several years after the strong earthquake.

### Key time points for vegetation recovery

The catastrophic Wenchuan earthquake caused surficial vegetation coverage in the mountainous areas to drop drastically. After the disturbance, the ecological equilibrium of the earthquake-affected zone fluctuated for a period. As shown in Fig. 6, the NDVI distribution in 2011 was lower than that in 2010 and it meant that the surficial vegetation coverage in the Mianyuan Basin was still weakening. Meanwhile, NDVI distribution in 2014 was greater than that in 2010 (as shown in Fig. 8b) and it continued to grow after 2014. This phenomenon illustrated that the surficial vegetation coverage of the Mianyuan River Basin in 2014 is no longer in the volatility period. Thus, the ecological balance fluctuation time of the earthquake-affected zone is 5 plus or minus 1 year.

After the volatility period, the surficial vegetation coverage in mountainous areas restored to the pre-earthquake state after several years of growth. Although the recovered surficial vegetation coverage cannot be exactly the same as that in the previous time, it can restore to quite a similar state. As shown in Fig. 8, the NDVI distribution curve on July 6, 2016 is roughly the same as that on April 30, 2008. Moreover, the surficial vegetation coverage on April 14, 2017 is basically the same as that on April 19, 2007. This suggests that the surficial vegetation of the earthquake-affected zone can basically recover to the pre-earthquake condition within 8 to 9 years after the earthquake.

### Overall evolution of surficial vegetation coverage

**Figure 13:**
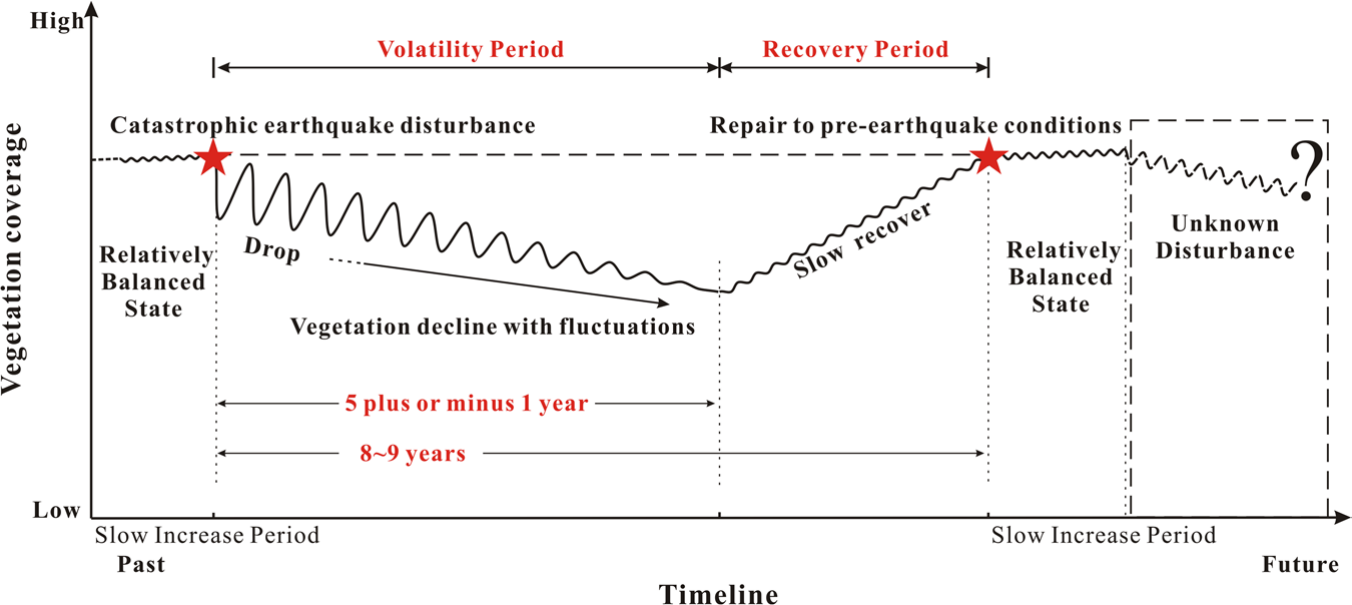
The overall evolution of surficial vegetation coverage under earthquake disturbance.

The ecosystem of nature is always in a state of volatility. Before the Wenchuan earthquake, the self-repairing process of the environment can moderate the ecological damage caused by small fluctuations immediately. However, after the disturbance of the strong earthquake, the ecological self-repair cannot rejuvenate the damaged environment immediately, and it will undergo a long-term evolution instead to restore the normal ecological fluctuation pattern before the earthquake. As shown in Fig. 13, before the strong earthquake, the mountain can moderate the ecological loss caused by slight disturbances in a short time because of its self-repair ability and thus the vegetation can stay in a relativity balanced state. Meanwhile, vegetation in the lower reaches of the basin and areas close to the river network shows slower recovery. After the strong earthquake, the vegetation coverage will go through volatility and recovery periods. A short time after the earthquake, the large-scale reduction in vegetation indicates wide-spread occurrences of shallow landslides. Middle and lower reaches and the area near the river network are more disturbed than high altitude areas. Moreover, the vegetation within 30 to 40 degrees and the near-fault area appears to be more susceptible to the disturbance. The earthquake will destroy the ecological balance of the mountainous area, making the whole area more susceptible to disturbances by other exogenic factors such as snowmelt and heavy rainfall. Therefore, during the volatility period, the vegetation of the mountainous area continued to decline. Vegetation in the middle and lower reaches, near river network and within 30 to 40 degrees recovers more quickly than that in other areas. After 5 plus or minus 1 year, earthquake-affected area enters the recovery period and vegetation starts to restore steadily. From the time of the earthquake to the end of the recovery period, it takes 8 to 9 years to restore the stage before the earthquake. After the recovery period, vegetation in the mountainous area has entered a slow increase period, and the surficial vegetation coverage will continue to grow slowly despite the slight disturbances unless there is a next strong earthquake.

## Conclusions

The catastrophic Wenchuan earthquake caused great disturbances to the ecology of the Longmenshan fault area and caused long-term effects on the environment. Remote sensing images were used to calculate surficial vegetation coverage in the earthquake-affected areas and the following conclusions can be drawn as follows: (1) Before the earthquake, vegetation in the lower reaches and near river networks grew better than that in other regions. (2) In the short period after the earthquake, the vegetation damage near the fault area and the river network is more serious than other areas. However, the areas with high surficial vegetation coverage were less disturbed by the earthquake. (3) The slopes at 30 to 40 degrees are more susceptible to earthquake disturbances but are easier to repair. (4) The vegetation near the fault is disturbed by the earthquake, but the vegetation restoration is independent of the fault. (5) The Wenchuan earthquake had a terrible impact on the vegetation in the southwestern mountainous areas of China. Ecological damage and repair in earthquake-affected areas underwent a volatility period of approximately 5 plus or minus 1 year. (6) In the 8–9 years after the earthquake, the mountain vegetation has basically recovered to the state before the earthquake.
